# Physiological tremor reveals how thixotropy adapts skeletal muscle for posture and movement

**DOI:** 10.1098/rsos.160065

**Published:** 2016-05-04

**Authors:** Carlijn A. Vernooij, Raymond F. Reynolds, Martin Lakie

**Affiliations:** 1 School of Sport, Exercise and Rehabilitation Sciences, University of Birmingham, Edgbaston, Birmingham B15 2TT, UK; 2 Institut des Sciences du Mouvement E.J. Marey (UMR 7287), Aix-Marseille Université and CNRS, 163 Avenue de Luminy, CP 910, Marseille 13009, France

**Keywords:** physiological tremor, mechanical resonance, thixotropy, posture, electromyography, muscle

## Abstract

People and animals can move freely, but they must also be able to stay still. How do skeletal muscles economically produce both movement and posture? Humans are well known to have motor units with relatively homogeneous mechanical properties. Thixotropic muscle properties can provide a solution by providing a temporary stiffening of all skeletal muscles in postural conditions. This stiffening is alleviated almost instantly when muscles start to move. In this paper, we probe this behaviour. We monitor both the neural input to a muscle, measured here as extensor muscle electromyography (EMG), and its output, measured as tremor (finger acceleration). Both signals were analysed continuously as the subject made smooth transitions between posture and movement. The results showed that there were marked changes in tremor which systematically increased in size and decreased in frequency as the subject moved faster. By contrast, the EMG changed little and reflected muscle force requirement rather than movement speed. The altered tremor reflects naturally occurring thixotropic changes in muscle behaviour. Our results suggest that physiological tremor provides useful and hitherto unrecognized insights into skeletal muscle's role in posture and movement.

## 1. Background

Human motor activity consists of periods of immobility, enlivened by periods of movement. How are these very different postural and dynamic roles addressed by the neuromuscular system? In many species, there is a division of skeletal muscle into tonic and phasic types with profound mechanical differences, for example amphibians [1], insects [2], birds [3] and reptiles [4]. Often the tonic types are arranged as bundles which parallel the phasic fibres, so that the same muscle can act in different ways depending on which bundle is activated. However, mammals, including humans, do not have specific muscle types for tonic or phasic behaviour. Instead, they draw on a common pool of skeletal muscle motor units, invariably recruited in order of increasing motor neuron size, to satisfy both posture and movement [5]. There are certainly mechanical differences between the initially recruited slow motor units and later recruited faster motor units. One study on human extensor hallucis brevis muscles showed contraction times with an extreme range of 35–98 ms [6]. These differences are actually rather slight, considering that posture may endure for periods of many seconds or minutes, whereas movements may be over in a fraction of a second. Thus, they seem more suited to generating movements of different contraction speeds rather than for the fundamentally opposed roles of movement and posture.

However, motor unit size and type are not the only determinants of muscle behaviour. There are profound mechanical differences between muscles when they are lengthening or shortening, and when they are static (or very nearly so). This difference is caused by muscular thixotropy, which can be defined as ‘the dependence of muscle stiffness on the history of length changes’. Thixotropic changes greatly alter the muscle's mechanical response to artificial stimulation and imposed movement [7–11]. While at rest, muscles become stiff and resistant to lengthening or shortening. If there is a change in muscle length which exceeds the minor elasticity inherent in cross-bridges, this ‘stiction’ falls to a low level, re-establishing itself rapidly as posture returns (the phenomenon was noted as early as 1929 by Denny-Brown [12]; he called it stationary rigidity). Thixotropic stiffening will ensure stability at rest, but will also permit unimpeded movement, thus enabling skeletal muscle to fulfil completely different postural and dynamic roles.

The implication is clear; muscle can be expected to act very differently when stationary and when moving. Such changes are easily demonstrated by *in vitro* experiments [13]. However, the changes in muscle behaviour *in vivo* are relatively inscrutable and have not been much studied. In a few recent studies, we have explored the altered relationship between the input to a muscle, measured as electromyography (EMG), and its output, measured as limb tremor (acceleration), as a consequence of muscle thixotropy (hand: [10,14] and finger: [11,15]). With movement, the peak in the tremor spectrum shifts to a lower frequency, whereas the shape of the EMG spectrum changes only slightly. The explanation is that the stiffness of the muscle changes (decreases), but its inertial load does not, thus altering the tuning of the muscle/limb oscillator and the way that it responds to the buffeting of motor unit firing. However, these studies separately examined posture and movement, and did not track the mechanisms underlying the transition between a period of rest and a period of movement.

In this paper, we describe the effect of transitions between posture and movement on muscle properties. Subjects used their middle finger to track a target that evolved from a static position into a very slow vertical sinusoidal movement before returning to a stationary position. We recorded surface EMG from the finger extensor muscle because it represents the neural drive to move the finger to overcome gravitational and elastic forces. We simultaneously recorded finger tremor as an indication of muscle output. We hypothesized the following: (i) tremor frequency will alter as a limb alternates between posture and movement; (ii) the frequency modulation can be parsimoniously explained by mechanical changes in muscle tissue and (iii) EMG magnitude will reflect the load but its frequency composition will not relate to tremor frequency.

Our results show that human physiological tremor, sometimes seen as little more than a curiosity, can illuminate the way in which muscle carries out its postural and dynamic duties and provide fundamental insights into the nature of posture and movement. Some of these findings have been briefly reported elsewhere [16,17].

## 2. Material and methods

### 2.1. Experimental volunteers

The experiment was carried out on 15 healthy, right-handed volunteers (23.7 ± 9.9 years old, three female) who gave written informed consent, and was undertaken in accordance with the declaration of Helsinki. Permission was obtained from the ethics committee of the University of Birmingham. None of the volunteers suffered from known neurological or muscular disorders. Participants were asked to refrain from exercising and not to consume alcohol or caffeine 24 h before participation.

### 2.2. Apparatus

The participants sat in a comfortable chair with the right forearm pronated and supported by a plastic curved rest. The hand as well as the index and ring finger were securely taped to a horizontal U-shaped aluminium support with a gap for the middle finger adjusted to fit individual subjects. This allowed for unhindered and isolated flexion–extension of the middle finger around metacarpophalangeal joint 3. A light duraluminium splint underneath the middle finger prevented movement at interphalangeal joints. The arm and the hand rest were individually connected to a heavy steel table by magnetic supports, so they could be optimally positioned.

**Figure 1.**
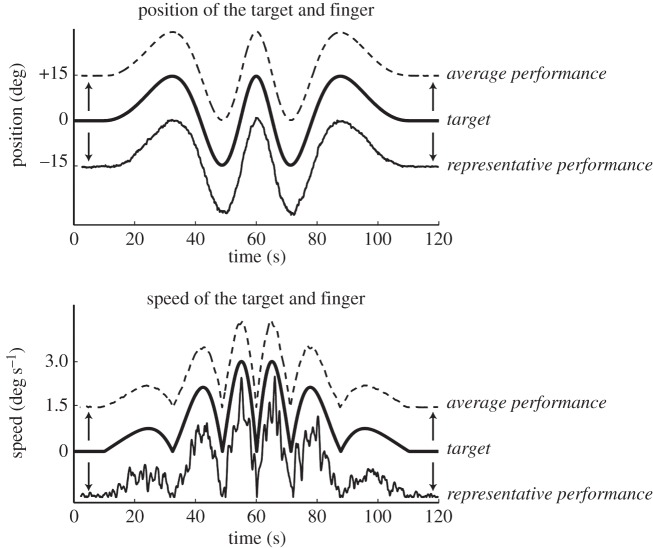
The target (thick black line), a representative performance (thin black line) and the average performance of all subjects/all trials (dashed) in the 120 s tracking task. Positive values represent an upward movement of the target and finger (extension). Note, for clarity, the traces have been vertically displaced so they do not superimpose.

A miniature three-axis accelerometer (model SCA3000, Active Robots, UK, 12.7 × 20.32 mm) was attached above the nail bed of the middle finger to measure its vertical acceleration. A retroreflective laser rangefinder (YP11MGV80, Wenglor Sensoric, Germany) was pointed at a white plastic reflective surface (approx. 2 × 3 cm) placed on top of the accelerometer to record vertical finger position. A computer screen approximately 1.5 m in front of the subject displayed finger position as a white cross and a computer-controlled target in the form of a red ball. The target was a stereotyped positional waveform which included transitions between posture and movement as shown in figure 1. Specifically, the target was initially static at an individually determined comfortable neutral (neither extended nor flexed) position of the finger. After 10 s, the target moved into a vertically orientated sinusoid with a frequency linearly rising from 0 to 0.05 Hz over 50 s (i.e. a chirp signal). The target then decelerated as a mirror image of this chirp and ended stationary in the neutral position for 10 s. The entire sequence occupied 120 s. The peak-to-peak amplitude of the waveform was approximately 30° of finger movement (15° extension (up) and 15° flexion (down)), which was well within the limits of the subjects’ comfortable range of motion. Participants were asked to track the target by keeping the white cross and red ball on the computer screen aligned. It was emphasized to the subjects that this was not a test of accuracy or precision and that they should remain as relaxed as possible. The maximum angular velocity required to track was 3 degree  s^−1^. Subjectively, this seemed a very slow movement, and this stereotyped task was easy to perform. Each subject repeated the task 10 times and refrained from moving their hand or fingers 10 s before the start of every trial. Surface EMG was recorded from the belly of the extensor digitorum communis muscle (m. EDC) with a Bagnoli system (Delsys Inc., USA).

### 2.3. Data analysis

**Figure 2.**
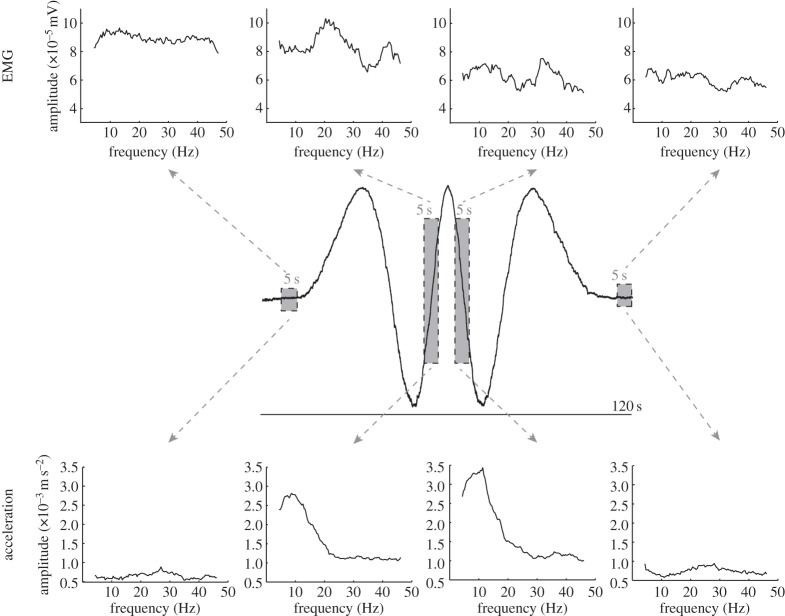
Frequency spectra of EMG and acceleration (tremor) at different phases of target tracking by a representative subject. Top: EMG frequency spectra. Middle: finger position. Bottom: acceleration frequency spectra. From left to right, spectra are shown for 5-s snapshots of static posture, maximum upward finger velocity, maximum downward finger velocity and static posture. The tremor spectra are relatively robust, whereas the EMG is much more variable. There is a considerable modulation of tremor frequency and size between posture and maximum velocity snapshots while there is no obvious direct correspondence with the related EMG spectra.

EMG was amplified ×1000. EMG, acceleration and positional data were sampled at 1000 Hz and digitized by an MC 6026 PCI card. Further analysis was carried out offline using custom Matlab scripts (MathWorks Matlab 2011a, USA). EMG was band-pass filtered (35–200 Hz, fourth-order Butterworth zero-phase-lag filter) and rectified. Acceleration signals were high-pass filtered (0.1 Hz, fourth-order Butterworth dual filter) to correct for artefactual modulation within the range of target frequencies. For one representative subject, the amplitude spectra of the finger acceleration and EMG were obtained by NeuroSpec software (v. 2.0, 2008) in order to display four 5 s snapshots at different phases of the 120 s target tracking (figure 2); static (second 5–10), maximum upward finger velocity (second 52.5–57.5), maximum downward finger velocity (second 62.5–67.5) and static (second 115–120).

Position, EMG and acceleration were then down-sampled to 100 Hz. To capture the non-stationary features of these signals (frequency and power) over the tracking movements, we calculated the wavelet transformation of EMG and acceleration. Wavelets depict the frequency components of a signal over time as if it were a frequency spectrum over a sliding time-window, therefore enabling us to characterize the frequency and power of EMG and acceleration associated with the different movement speeds and positions of the finger. The mean wavelet power was calculated using a continuous wavelet transform (CWT) ([10,18]; for a tutorial, see [19]). The CWT scales and translates a mother wavelet shape at each infinitesimal time step of a continuous signal. The wavelet coefficient *C* of signal *s* over time *t* is calculated as: $C_{a,b}=\int \;s(t)(1/\sqrt{a})\Psi *((t-b)/a)\;dt$, where *Ψ* is the mother wavelet, *a* is the frequency scale of *Ψ*, *b* is the position of *Ψ* and * is the complex conjugation. We used a complex Morlet mother wavelet with a bandwidth *f*_b_ of 1 Hz and a central frequency *f*_c_ of 1.5 Hz, so that $\Psi (x)=\;(1/\sqrt{\pi f_{b}})\;e^{2t\pi f_{c}x}\;e^{-x^{2}/f_{b}}$. The wavelet was scaled to correspond to a frequency range of 5–37.5 Hz. Wavelet analysis allowed us to examine the peak power and frequency of acceleration and EMG over the 120 s duration of the tracking trial. Note that ‘power’ here is relative, as the wavelet analysis transforms the data. Absolute velocity (speed) of the finger was obtained by differentiating the positional signal (second-order Savitzky–Golay filter, time window = 151 data points).

### 2.4. Statistics

**Figure 3.**
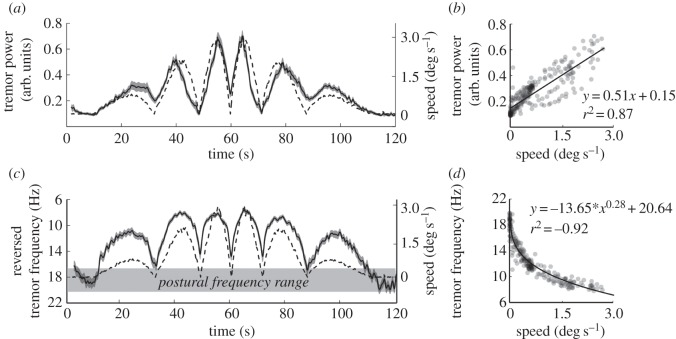
(*a*) Finger acceleration (tremor) power (arbitrary units, solid line ± s.d.) as depicted by wavelet analysis is shown alongside movement speed (dashed line). (*b*) Tremor power plotted as a function of movement speed. A linear regression provides a satisfactory fit, although it may misrepresent the relationship when movement is slow. (*c*) Tremor frequency (solid line ± s.d.) as depicted by wavelet analysis and movement speed (dashed line). The postural tremor frequency range is also indicated (details in text). Note that the scale for frequency runs from 6 to 22 Hz and is reversed to emphasize the striking correspondence with movement speed. (*d*) Tremor frequency plotted as a function of movement speed. A power function provides an excellent fit.

**Figure 4.**
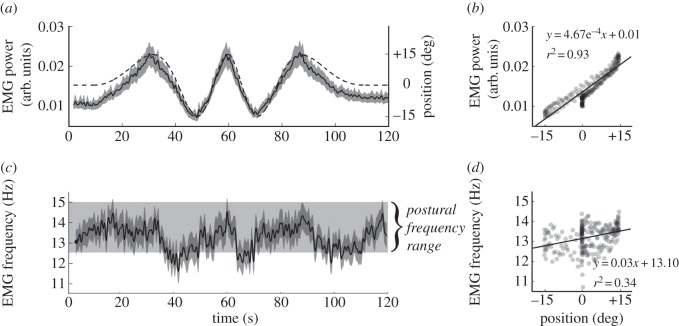
(*a*) EMG power (solid line ± s.d.) as depicted by wavelet analysis and movement speed (dashed line). (*b*) EMG power plotted as a function of finger position. A linear regression provides an excellent fit. (*c*) EMG peak frequency as depicted by wavelet analysis. The postural frequency range is also shown (details in text). Note that the modulation of frequency is different when the finger is rising and when it is falling. Also note that the EMG peak frequency does not correspond to the tremor frequency as seen in figure 3. (*d*) The relationship of EMG peak frequency to finger position. The regression line shows that EMG frequency is on average 0.81 Hz greater when the finger moves from −15 to +15°, but the relationship is weak and the coefficient of determinance (*r*^2^) is low.

We wanted to know whether, and at which time point during the transition between posture and movement, the frequency of acceleration and its neural drive would significantly change. Therefore, the 95% confidence interval (CI) was calculated for the peak frequency of acceleration and EMG over time. For both variables, the frequencies covered by the CI over the last 5 s of the two static periods (at the start and end of the movement) were designated the ‘postural’ frequency range (figures 3*c* and 4*c*). We then measured the time when the limits of the CI during the tracking movement would exceed this range, of which the first and last time points were used to signify a borderline finger movement that signifies a change in frequency. Wavelet data as well as finger position and finger velocity were averaged over trials and subjects and finally smoothed (moving average with time constant = 0.4 s).

## 3. Results

### 3.1. Tracking task performance

Figure 1 shows the position of the target waveform with the average tracking performance and a representative tracking performance. The associated speed of target and finger are also plotted. It is clear from this figure that the two were, on average, very well matched. This was to be expected for a slow, repetitive and predictable target movement [20]. Small differences between the target and the response are clearly visible in the position and speed records for representative single trials. Figure 2 shows the frequency spectra for EMG (top) and tremor acceleration (bottom) of one representative subject at four phases of the tracking; static, maximum upward velocity, maximum downward velocity and static, respectively. The corresponding finger position is shown also (middle). Whereas there is a large modification of the tremor spectra in both size and frequency between posture (left and right panels) and movement (middle panels), the differences in EMG are much smaller between the different tracking phases.

### 3.2. Relationship between acceleration (tremor) and finger movement

Figure 3 illustrates the evolution of wavelet-derived tremor (acceleration) power (figure 3*a*) and frequency (figure 3*c*) over time during the tracking task (average ± s.d.). The s.d. is very small and hard to detect, depicting the small variance between subjects and trials. There is a striking resemblance between the two acceleration variables and the superimposed average finger speed. Acceleration exhibits an increase in power and decrease in frequency almost immediately after movement onset, and covers a wide range of frequencies. When finger speed exceeds 0.15 degree s^−1^ the frequency drops below the postural frequency bands (figure 3*c*; CI = 16.6–20.6 Hz) shown in grey. At the fastest speed (3 degree s^−1^), acceleration frequency reduces to 7.8 Hz. When the finger speed drops towards zero for short periods, the tremor frequency just re-attains the postural frequency band. When the ongoing movement is faster, it fails to re-attain the postural frequency band. Only when the finger is stationary for some time towards the end of the experiment does the frequency definitely re-enter the postural band. The right-hand panels of figure 3 display the relationship between finger speed, and acceleration power (figure 3*b*) and frequency (figure 3*d*) averaged over 0.5 s bins. There is a positive linear relationship between power and speed (average individual *r*^2^ = 0.64 or *r*^2^ = 0.87 for mean signals) and negative power-relationship between frequency and speed (average individual *r*^2^ = −0.41 or *r*^2^ = −0.92 for mean signals).

### 3.3. Relationship between EMG (neural input) and finger movement

There is a stark contrast between acceleration and EMG, both in terms of the overall range of frequencies covered and the way that they respond to movement. Figure 4*a* shows EMG wavelet power almost exactly superimposed upon finger position, and the two signals are strongly linearly related (figure 4*b*; average individual *r*^2^ = 0.69 or *r*^2^ = 0.93 for mean signals). Not surprisingly, more EMG in the extensor muscle is recorded when the finger moves to a correspondingly higher (more extended) position. Figure 4*c* shows that, in complete distinction to peak acceleration frequency described above, EMG frequency during movement remains mostly within the postural frequency range (CI = 12.5–15.1 Hz). This relatively small range covers the middle frequencies of those covered by the acceleration (7.8–20.6 Hz). Relatively, steep drops in EMG frequency occur when the finger moves downwards, but only once is there a short period where the frequency significantly (i.e. average ± CI) decreases below the postural range. Overall, there is a slight increase in EMG frequency with higher finger positions (figure 4*d*; average individual *r*^2^ = 0.06 or *r*^2^ = 0.34) for mean signals.

### 3.4. Power–frequency relationship within EMG and acceleration

**Figure 5.**
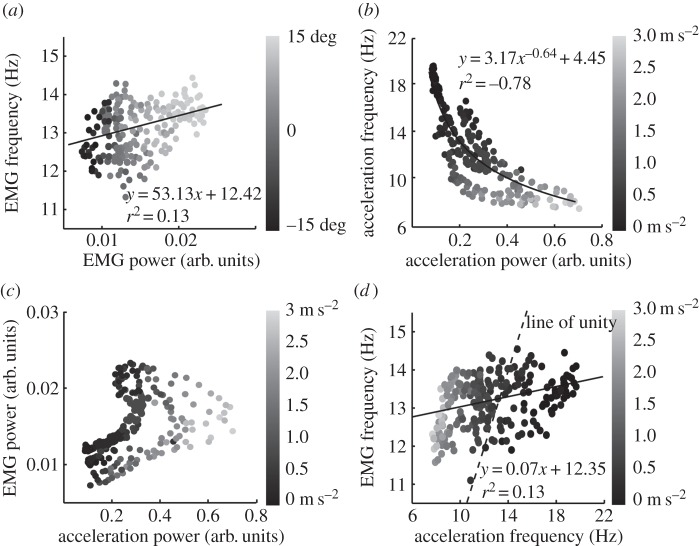
In (*a*), the greyscale represents finger position. In (*b*–*d*), it represents finger speed. Black always represents the lowest values. (*a*) EMG peak frequency as a function of EMG power. The highest finger positions are associated with the greatest EMG power and the highest peak frequencies. (*b*) Acceleration frequency as a function of acceleration power. The highest finger speeds are strongly associated with the greatest acceleration power and the lowest frequencies. (*c*) EMG power as a function of acceleration power. The highest finger speeds are associated with the greatest acceleration power, but there is no strong relationship between speed and EMG power or acceleration power and EMG power. (*d*) The relationship between EMG peak frequency and acceleration frequency. The highest finger speeds are associated with the lowest frequency of both acceleration and EMG but the two variables alter by very different amounts as indicated by the line of unity (dashed line).

The average relationship between power and frequency for EMG and for acceleration is shown in figure 5. In each panel, finger kinematics are represented by the use of greyscale (finger *position* in *a*; finger *speed* in *b–d*), where a fade to black implies lower values. Figure 5*a* displays a significant positive relationship between EMG power and frequency. In general, as EMG power increases, so does its frequency (*r*^2^ = 0.13, *p* < 0.01), but there is considerable variability. The superimposed greyscale captures the much stronger positive relationship between EMG power and *finger position* (also shown in figure 4*b*). Figure 5*b* shows a significant *negative* relationship between power and frequency of finger acceleration. The largest drop in frequency occurs when the finger begins to move, i.e. at low finger speeds where the acceleration is small (denoted by darker black dots). A power-curve fit gave a strong correlation of *r*^2^ = −0.78 (*p* < 0.01).

As any acceleration *has* to be a consequence of some neural input, whether broad-band or frequency-specific input, we also studied the relationship between EMG and acceleration by correlating their power and frequency. Figure 5*c* shows an expected tendency for more acceleration with increased EMG power, but the strength of this correlation is weak (*r*^2^ = 0.18, *p* < 0.01) and the relationship is complicated; acceleration power can be small when EMG is both high and low. This corresponds to the situation when the finger is stationary at its top or bottom position, respectively. The greyscale shows the previously described positive correlation between finger speed and acceleration power (seen in figure 3*b*). Figure 5*d* shows no strong modulation of acceleration frequency with EMG frequency. Although a significant positive linear relationship does exist (solid line; *r*^2^ = 0.13, *p* < 0.01), this line is very different from the line of unity (dashed line) indicating that there is no direct causal relationship. Again, the strong negative relationship between acceleration frequency and finger speed is obvious in the greyscale.

## 4. Discussion

Here, we examined the behaviour of physiological finger tremor during transitions between posture and movement. This is important, because tremor during (slow) movement has been claimed to be fundamentally different from postural tremor [21]. However, although the relationship of the phase of tremor with rapid voluntary movement has been studied before, the way in which postural tremor at rest evolves into the kinetic tremor of movement, and vice versa, has not been characterized. Our results show that there are predictable (large) alterations in the power and frequency of tremor as posture becomes movement. These alterations are not shown in the concomitantly recorded EMG which drives the muscle. There has been a tendency in the literature to discuss ‘10 Hz tremor’ as though it provides ‘a window into the operation of the nervous system’ [22]. What this study demonstrates is that systematic and quite large alterations in tremor frequency can be generated by movement, and it is not easy to explain these changes as being produced by a neurogenic oscillator. On the other hand, it is quite easy to attribute them to mechanical causes. We discuss whether alterations in the stiffness of the muscles, which occur during movement, produce the altered tremor power and frequency and whether physiological tremor can provide practical and previously unrecognized insights into the stiffness of the musculature.

### 4.1. Acceleration and EMG changes with movement

The power and frequency of physiological finger tremor (measured as acceleration) are strongly modulated by the *speed* of finger movement, with faster movement being associated with progressively larger and slower oscillations. The change is obvious in the initial stages of movement; acceleration frequency drops substantially and power increases as soon as movement begins (figure 3). This is not true for the associated EMG power which correlated nearly linearly with finger *position* (figure 4*a,b*). Throughout the 120 s trial, the EMG frequency stays within a relatively narrow range (12.5–15.1 Hz), which is situated neither at the high nor low end of the range of acceleration frequencies (7.8–20.6 Hz; figures 3 and 4). There is no simple relationship between the frequency of the acceleration (which alters a lot) and the frequency of the EMG (which alters only a little). This suggests that finger tremor under normal, healthy circumstances might not necessarily be generated by a specific neural input. This gives rise to an important question: are the characteristics of finger tremor determined by the mechanical properties of the limb?

The strong correlation of extensor EMG power with finger position (figure 4) was entirely expected because it reflected the increased muscle force required to raise the finger. The activity of the main flexor muscle is not reported here as it hardly exceeded the background level and any activation present was erratic. The maximal velocity (which was attained slowly) was only 3 degree s^−1^, which is extremely slow. Therefore, for the speeds that we studied the braking forces were almost certainly passive and mechanical. The downward force was mainly applied by elastic forces in the flexor muscles owing to passive stretch or, more likely, gravity. Some modulation of EMG frequency was also anticipated. For several reasons, there is a generalized increase in EMG frequency as force is increased (reviewed by [23]), for instance, because EMG will be modulated according to rate coding. EMG frequency drops abruptly as the finger commences downwards movement (flexion) and rises more slowly and progressively as the finger is extended (figure 4*c*). This is probably owing to the different efficiency of the extensor muscle as it acts concentrically and eccentrically. In addition, some EMG frequency modulation could have been caused by measurement artefacts owing to a change in muscle fibre orientation relative to the EMG electrode [23]. Independent of its cause, and more importantly here, there were no corresponding hysteretic effects in tremor acceleration frequency (figure 3*c*). This divergent behaviour is additional simple evidence against the idea that physiological tremor frequency is mainly a reflection of the underlying frequency of the EMG.

### 4.2. The resonant nature of physiological tremor

In general, physiological finger tremor is described as being composed of a number of acceleration frequency components of distinct origins. Often a combination is proposed of two centrally determined modes of oscillation (a postural tremor generator and a kinetic tremor generator) or a combination of mechanical limb properties and neural oscillations of central origin [24,25] or reflex origin [26,27]. However, our previous research suggested that all frequencies reported in finger tremor could be economically explained by resonance [11,15,28] which differs in frequency depending on the state of the musculature. When the finger and muscle do not move, muscle stiffness is high, thereby generating a high-frequency component greater than 20 Hz. On the other hand, when muscle moves its stiffness is low and generates a frequency component approximately 8 Hz. (This concept is further discussed below.) This study adds to that research by showing clearly that acceleration power and frequency are modulated by the speed of movement. The smooth modulation and lack of substantial change in the EMG suggest there is a *single* peripheral determinant of frequency that adjusts its properties with movement.

### 4.3. Thixotropic stiffening of muscle raises resonant frequency

The finding that finger tremor is modulated by the speed of finger movement ties in neatly with a mechanical resonance origin. It is known that extrafusal and intrafusal muscular stiffness is dependent on the recent history of movement [8,29]. When muscle moves, its stiffness reduces greatly, for example by a factor of approximately 15 in the human calf muscles [30]. Although still currently debated in the literature, conceivably the underlying mechanism consists of the elastic characteristic of cross-bridges and the number of actin–myosin attachments [8,13,31,32] with the additional contribution of unfolding of gap–filament proteins like titin [33,34]. Whatever the mechanism, during posture, muscle stiffness is high. During movement, the postural short-range elastic stiffness (SREC) is transformed into a smaller, approximately constant, frictional resistance [31]. If a steady posture is subsequently maintained, stiffness will slowly regenerate over the following seconds. This phenomenon, known as thixotropy, makes muscle stiffness very strongly dependent on length changes.

With movement, there is substantially decreased muscle stiffness and thus a decreased resonant frequency. The muscle length, resulting from a different finger position, is not the determining factor, because thixotropic stiffening occurs at all muscle lengths [13]. In a reduced preparation (single muscle or single muscle fibre), the transition from SREC to frictional behaviour is very abrupt [13]. In a limb which is controlled by different synergistic and antagonistic muscles and very many muscle fibres, the transition will be expected to be less abrupt and this is what we report here. However, it is clear that the greatest reduction in frequency occurs for very small movements (figure 3*d*). This behaviour is entirely consistent with a reduction in stiffness once a very small range of movement is exceeded. Hill [31] described the range of the SREC *in vitro* as 0.2% of muscle length. Several studies have shown that *in vivo* the required muscular movement to exceed the SREC is very little [8,35]. The tiny movements associated with ‘posture’, i.e. the tremor itself, are accommodated within the SREC range. In contrast, even with very slow movements, the SREC is continually exceeded, and the frequency remains lower than the postural range. This is exactly what we find here (figure 3*c*).

### 4.4. The implications for physiological tremor recording

These results emphasize the importance of controlling very carefully the conditions under which tremor is recorded. Often, twin peaks in the postural tremor spectrum are discussed in the literature, e.g. [36,37], but this does not necessarily imply they occur simultaneously as is always assumed. When holding the finger extended the muscle stiffness and resonant frequency will be high. However, an occasional small postural adjustment will elicit a temporary drop in muscle stiffness, which will generate a temporary low resonant frequency. A Fourier transform displays the average frequency spectrum of a tremor recording, typically lasting 30–60 s. If small postural adjustments are interspersed with posture, both high and low frequencies will be present in the tremor FFT, both produced by the same mechanism. If the Fourier transform of postural tremor is taken over a few seconds only (as in figure 2), the low frequency peak may be absent.

### 4.5. What is posture, what is movement?

Hill [31] and others have shown very clearly that the threshold of the SREC is one of position. The SREC will re-exert itself at any muscle length *if the muscle is allowed to rest there for sufficient time*. Rest, presumably, is not absolute, but is in reality a period where velocity is very low. There is no such thing as a fixed position, because there is always minor movement, positional drift and tremor. Figure 3 shows that the confidence limits of postural tremor frequency are exceeded when movement speed reaches a speed of 0.15 degree s^−1^. Although this is by definition a movement, its speed is so slow that it is not greatly above the threshold for detection if it was to be passively applied [38]. The postural frequency range is only confidently re-entered when the finger is stationary for some time (110 s onwards; figure 3*c*). This strongly suggests posture and movement are quantitatively, rather than qualitatively, different.

### 4.6. Is muscle thixotropy the basis of posture and movement?

The question of the distinctiveness of movement and posture is an old one, with some authorities, but not all, suggesting that specialized neural pathways are involved [39]. Our results do not answer that question. However, it is undeniable that in man the ‘final common pathway’ for both movement and posture is a single set of skeletal muscles which must satisfy the very different requirements of both roles. Muscle stiffness increases greatly as muscle lengthening or shortening ceases, and our results suggest that tremor provides a window into this natural behaviour. They show that as muscle transitions from lengthening or shortening to posture, tremor frequency increases, reflecting an increase in muscle stiffness and resonant frequency. This thixotropic stiffening of muscle is due to the reformation of actin–myosin attachments in combination with non-cross-bridge mechanisms. It is likely that this has a stabilizing influence, making it easy to minimize speed for long periods of time. By definition, this is posture. Man-made servo systems for controlling manufacturing processes, elevator systems, valves and the like, very commonly incorporate an electromechanical brake, so that a static position can be economically maintained. Thixotropic muscle stiffening, which permits the economical and effective control of posture and movement by a common actuator, may be nature's version of the brake. Meanwhile, with little movement, thixotropic loosening induces very large reductions in muscle stiffness (up to 15-fold; [35]), allowing unimpeded and rapid motion. An example where the functional impact of thixotropy is clear is when raising a gun to shoot a target. This task generates a conflict between movement and stability. The marksman's muscles are not very stiff when the gun is raised, to enable a large angular momentum. His muscles then get very stiff when he takes aim to maintain joint orientation. The transition in muscle stiffness solves the conflicting requirements of flexibility and speed during fast action, and accuracy and stability at rest. Thus, skeletal muscle is optimized for not only posture, but also for movement.

## Data accessibility

All raw data are available through the Dryad repository: http://dx.doi.org/10.5061/dryad.r1j0s.
